# Total Sleep Deprivation Followed by Bright Light Therapy as Rapid Relief for Depression: A Pragmatic Randomized Controlled Trial

**DOI:** 10.3389/fpsyt.2021.705090

**Published:** 2021-08-30

**Authors:** Michael Ioannou, Zoltán Szabó, Mats Widmark-Jensen, Georgios Vyrinis, Christopher Karlsson, Steinn Steingrimsson

**Affiliations:** ^1^University of Gothenburg, Sahlgrenska Academy, Institute of Neuroscience and Physiology, Gothenburg, Sweden; ^2^Region Västra Götaland, Psykiatri Affektiva, Department of Psychiatry, Sahlgrenska University Hospital, Gothenburg, Sweden; ^3^Region Halland, Varberg's Hospital, Anaesthesia and Intensive Care, Varberg, Sweden

**Keywords:** depression, sleep deprivation, phototherapy, chronotherapy, inpatients, pragmatic randomized clinical trial

## Abstract

**Background:** Total sleep deprivation (TSD) combined with bright light therapy (BLT) has been suggested as a valuable add-on to standard treatment for rapid relief of depression. However, there is a lack of randomized controlled trials in real-life clinical settings. The aim of this pragmatic randomized clinical trial was to investigate the effectiveness, acceptance, and feasibility of TSD combined with BLT as add-on to standard treatment for depression in a real-life clinical setting.

**Methods:** Thirty-three inpatients were randomly assigned to either: a) an intervention group receiving a single-night TSD followed by 6 days BLT (10.000 lux, 30 min/day) as add-on to standard treatment; or b) a control group receiving a short sleep-hygiene consultation in addition to standard treatment. The follow-up period was 1 week.

**Results:** No statistical differences were found in response rates, reduction of depressive and insomnia symptoms, length of stay, readmission rate, and clinical improvement. Both groups reported positive experiences toward the received treatment with low drop-out rates.

**Conclusions:** One-night TSD followed by BLT was not effective as a rapid relief for depression at 1-week follow-up; however, the treatment was feasible and well-tolerated.

## Introduction

The rapid relief of depressive symptoms is paramount in treating acute depressive states, especially when risk for suicide is high or other indications for psychiatric hospitalization are present (1, 2). There is growing evidence for novel, rapidly-acting antidepressant treatments such as ketamine (3, 4), scopolamine (5), brexanolone (6), and accelerated transcranial magnetic stimulation (7). Chronotherapy has also been suggested as a valuable add-on to current antidepressants for the rapid improvement of mood and sleep (8). The modus operandi of chronotherapeutic interventions is based on sleep manipulation and utilization of zeitgebers, in particular light (9).

Total sleep deprivation (TSD) or wake therapy (i.e., intentionally staying awake during one or several nights with recovery sleep in between) is the main method for manipulating sleep timing and duration. Several studies have reported a rapid but transient alleviation of depressive symptoms directly after TSD (10). By combining single or repeated TSD with other chronotherapeutic interventions such as bright light therapy (BLT), sleep time stabilization (STS) and sleep phase advance (SPA), the primary effect of TSD might be sustained (11, 12). Although TSD has been investigated as an antidepressant treatment in four decades, only a handful RCTs (with diverse protocols and results) have been conducted leading to uncertainty over the efficacy of the treatment (10, 13, 14). Moreover, suicidality and self-harm are commonly exclusion criteria in controlled trials on chronotherapy, affecting external validity (10).

There is namely a concern that the observed effectiveness of treatments tested in clinical trials may not reflect their actual effectiveness in usual practice (15). Pragmatic trials are prominent for examining the effectiveness of interventions in a real-world clinical setting and covering the full spectrum of the population to which the intervention will be applied (16). In contrast to explanatory studies, the study design is often simple with fewer endpoints and more patient-centered outcomes (17). Apart from effectiveness, the feasibility and acceptance of interventions can also be examined (18). Tools such as the pragmatic explanatory continuum indicator summary (PRECIS-2) have been developed to facilitate pragmatic design in clinical trials, thus highlighting the importance of pragmatic trials in the research field (19).

Despite promising results, the implementation of combined chronotherapy in daily clinical practice remains limited (20, 21). One-night TSD followed by BLT has been suggested as an effective and well-tolerated chronotherapeutic protocol but it has not been evaluated in controlled trials (14). However, two open-label studies showed promising results of combined chronotherapy in depressed suicidal inpatients (22, 23). Based on the current literature of TSD, there is a need for pragmatic randomized clinical trials (RCTs) of 1-night TSD followed by BLT as add-on treatment for rapid alleviation of depressive symptoms.

The main aim of this study was to examine the effectiveness, acceptance, and feasibility of total sleep deprivation followed by 6 days BLT as adjunctive treatment for inpatients with depression. Therefore, the null hypothesis (H_0_) in our analysis was the mean additive effect of TSD/BLT is equal to the mean additive effect of short sleep-hygiene consultation.

## Materials and Methods

### Study Design

The study was a 1-week RCT with two groups: an intervention group which received 1-night TSD followed by 6 days BLT as adjunctive treatment to standard treatment (TSD/BLT group) and a control group which received a short sleep hygiene consultation in addition to standard treatment. A pragmatic perspective was used during study designing, scoring 42/45 using the PRECIS-2 tool (19) (Table 1). The trial was pre-registered (ClinicalTrials.gov identifier NCT02503124). The study follows the CONSORT criteria for reporting RCTs with pragmatic design (24).

**Table 1:** PRECIS-2 scores for trial domains.

**Domain**	**Score**	**Rationale**
1. Eligibility criteria	4	Pregnancy was an exclusion criterion
2. Recruitment path	5	Pre-screening by clinicians after admission
3. Setting	5	No extra personnel, recourses, or costs
4. Organization intervention	5	Identical organization to usual care
5. Flex of experimental intervention – delivery	4	Recently admitted inpatients only
6. Flex of experimental intervention – adherence	4	No extra personnel but availability of social and physical activities during night
7. Follow-up	5	Very pragmatic approach with no more than usual follow-up
8. Outcome	5	Self-ratings, patient experiences as outcome
9. Analysis	5	Intention-to-treat analysis
Total	42	

*PRECIS-2 scoring: 1 = very explanatory, 2 = rather explanatory, 3 = equally pragmatic/explanatory, 4 = rather pragmatic, and 5 = very pragmatic (for more information see https://www.precis2.org/). PRECIS-2, Pragmatic Explanatory Continuum Indicator Summary, version 2*.

### Changes to the Study Design

According to the initial trial design, all study participants were to be followed up at 10 weeks after hospital discharge for evaluation of depressive and insomnia symptoms as well as medication use. Due to the high rate of losses to follow-up among the first 10 participants, no follow-up visits were planned for the remaining participants. Furthermore, the trial was ended during the spring of 2020 as recruitment during the COVID-19 pandemic was impossible due to local restrictions.

### Study Setting

The study was conducted in four psychiatric wards at the Sahlgrenska University hospital (Sweden) from August 2015 to February 2020. The wards consist of 14–15 beds for patients with acute psychiatric problems with one attending specialist in psychiatry per ward. Three psychiatric wards were dedicated to general psychiatric services and one was specialized for patients with bipolar disorder. During the study period, two of the general psychiatric units were closed down due to economic reasons. Only one ward had used TSD prior to the study (25).

### Participants

The inclusion criteria were: voluntarily admitted inpatients aged 18–65 years; not admitted at the inpatient unit for >4 days at the time of screening; Mini International Neuropsychiatric Interview (MINI) diagnosis of current depressive episode and clinically assessed as the main clinical diagnosis; ability to speak and understand Swedish; and use of a stable mood-stabilizing treatment regimen prior to hospitalization (if bipolar depression). Exclusion criteria were: psychotic symptoms on admission or screening; planned or undergoing electroconvulsive therapy; pregnancy; symptoms or signs of drug or alcohol withdrawal; eye disorders; porphyria; and epilepsy.

### Study Procedures

#### Recruitment and Screening

Newly admitted patients with depression were informed about the ongoing study and pre-screened by their physician. The inpatients could thereafter receive further oral and written information by the study investigators. Screening and obtaining informed consent from eligible participants were performed by the study investigators. Apart from the diagnostic assessment made by the patient's physician, the diagnosis of depression was confirmed using MINI during the screening process. All participants filled the following self-rating scales prior to randomization: Alcohol Use Disorders Identification Test; Drug Use Disorders Identification Test; and Morningness-Eveningness Questionnaire (MEQ), a 19-item self-assessment scale which assesses the individual's diurnal rhythms and underlying chronotype (26). The MEQ total score ranges from 16 to 84, with higher scores indicating stronger morning preference. Moreover, an algorithm for timing BLT based on the MEQ score has been developed, starting the treatment about 8.5 hours after the estimated melatonin onset (14).

#### Randomization

The participants were allocated to the two similar sized groups using block-randomization of 10 patients using an online randomization algorithm. Sealed, opaque, sequentially numbered envelopes were opened by the investigator following inclusion, which informed the participant about their allocation. Blinding was not possible due to the nature of treatment.

### Study Interventions

#### Intervention Group

The intervention group (TSD/BLT group) received the following chronotherapeutic protocol as add-on to standard treatment:

TSD: Participants stayed awake for 33–36 hours (i.e., 1 night), starting on the same day as recruitment or the next day. The ward staff was encouraged to support the patients during the sleep deprivation (wakefulness) phase. Adherence strategies included social and physical activities (e.g., games, socializing, watching TV, short walks in the courtyard, and availability of food and drink during the night). During the TSD phase, the use of sleep medication and/or benzodiazepines was not allowed. Patients were recommended not to sleep until 8 pm the night following TSD.

BLT: Patients received 30 min of light therapy for 6 mornings, starting after recovery sleep from TSD. The optimal timing was individually based on the MEQ total score (27). A daylight lamp with 10,000 lux white light (Philips EnergyLight HF3419/01 or HF3319/01, Netherlands) was used. BLT was administrated in the patient's room by the ward staff after a short introduction by the investigators based on the manufacturer's instructions for proper use.

#### Control Group

The control group received a short sleep hygiene consultation in addition to standard treatment. It consisted of one session of cognitive behavioral treatment-based psychoeducation on sleep hygiene (28). The main focus was mapping current sleep habits, proposing sleep hygiene tips, and discussing their implementation during the hospitalization period. The content of the sleep hygiene consultation was standardized but emulated regular clinical advice. The consultation was given by the research investigators and lasted 25–45 min. The baseline scores of the self-rating scales were principally used as a starting point for the consultation.

#### Standard Treatment

Standard treatment was individualized and consisted of regular medical and psychiatric treatment. Changes in medication were allowed during the study period. None of the participants received inpatient psychotherapy or standardized behavioral activation.

#### Implementation of the Intervention at the Psychiatric Ward

The ward staff was informed regularly about the study's rationale and procedures during workplace meetings. Prior to every administration of combined chronotherapy, the instructions regarding TSD and BLT were repeated to the night staff. Three residents in psychiatry were recruited as sub-investigators in the study. The investigators were available to answer questions from the ward staff during the intervention period. No extra resources or personnel were added to the usual care settings. No further standardization of the intervention was conducted beyond the clinical instructions to the ward staff.

### Outcome Measures

#### Primary Clinical Outcome

The primary outcome was the clinical response rates defined as ≥50% decrease in the baseline Montgomery-Åsberg Depression Rating Scale - Self Assessment (MADRS-S) score 1 week after enrollment. In this study, the self-rated version was preferred in accordance with common clinical practice in Swedish psychiatric wards. One week was deemed as a relevant follow-up duration when evaluating rapid inpatient treatment in the hospital setting where the study was conducted (23).

#### Secondary Clinical Outcomes

The following were measured as secondary outcomes:

- Clinical remission rates (MADRS-S total score ≤ 10).
- Reduction in depressive symptoms measured with MADRS-S.
- Reduction in insomnia symptoms measured using the Insomnia Severity Index (ISI), a 7-item self-report questionnaire that assesses daytime and nocturnal symptoms of insomnia (29). The total score ranges from 0 to 28, with a score of ≥ 8 indicating clinically relevant insomnia symptoms (30). A 1-week recall period was used as previously reported (31).
- Number of participants with an ISI score reduction ≥ 50%.
- Change from baseline in the severity of patient's illness and response to treatment measured with the Clinical Global Impression (CGI) scale (32).
- Length of hospital stay.
- Readmission rate.
- Rate of revisit to the psychiatric emergency room.

#### Self-Reported Patient Experiences

A two-version questionnaire was developed for evaluation of acceptance of the received interventions (TSD/BLT and sleep hygiene consultation) after treatment completion. It consisted of a 7-item Likert scale which assessed the participant's experienced cognitive and emotional responses to the interventions. In the 7 statements of the Likert scale a 5-point symmetric agree-disagree scale was used (from 1 = “do not agree at all” to 5 = “fully agree”). The questionnaire captures important components of the theoretical framework of acceptance, e.g., affective attitude, ethicality, burden, perceived effectiveness, and self-efficacy (33).

#### Complications

Change in agitation levels during TSD were measured using a modified 5-item version of the Positive and Negative Syndrome Scale (PANSS-5, excitement, hostility, tension, uncooperativeness, and poor impulse control) (34). Thus, agitation levels were measured in the morning before and after the night of TSD. Adverse events were assessed regularly by the investigators during the whole study period. Furthermore, a question on adverse events with a free text option was asked at treatment completion.

### Statistics

A power analysis based on 50% response in the intervention group and 15% in the control group showed 54 patients would be needed, given a probability level of 0.05 and a power of 0.80 (23, 35). Descriptive statistical comparisons of frequencies and means by group were utilized to describe population characteristics. Sociodemographic variables were compared using Student's *t*-test or chi-square tests. Homogeneity of variance was tested by Levene's test. Treatment effects (remission, response, MADRS-S, ISI, and CGI) between groups were analyzed with Student's *t*-test or Fisher's exact test on using the intention-to-treat principle. Paired *t*-tests were used for pre-post measures in each group and Cohen's d type effect size was calculated for each measure. Kaplan-Meier survival plots and log-rank tests were performed for evaluating differences between groups regarding time to revisit to the psychiatric emergency room and readmission to psychiatric ward 30 and 90 days after discharge. The significance level was set at *p* < 0.05. The internal consistency reliability of the questionnaire for self-reported patient experiences was measured in terms of Cronbach's (alpha) coefficient and inter-item correlations. Analysis was conducted using IBM SPSS Statistics 26.

## Results

### Participant Flow and Characteristics

**Figure 1:**
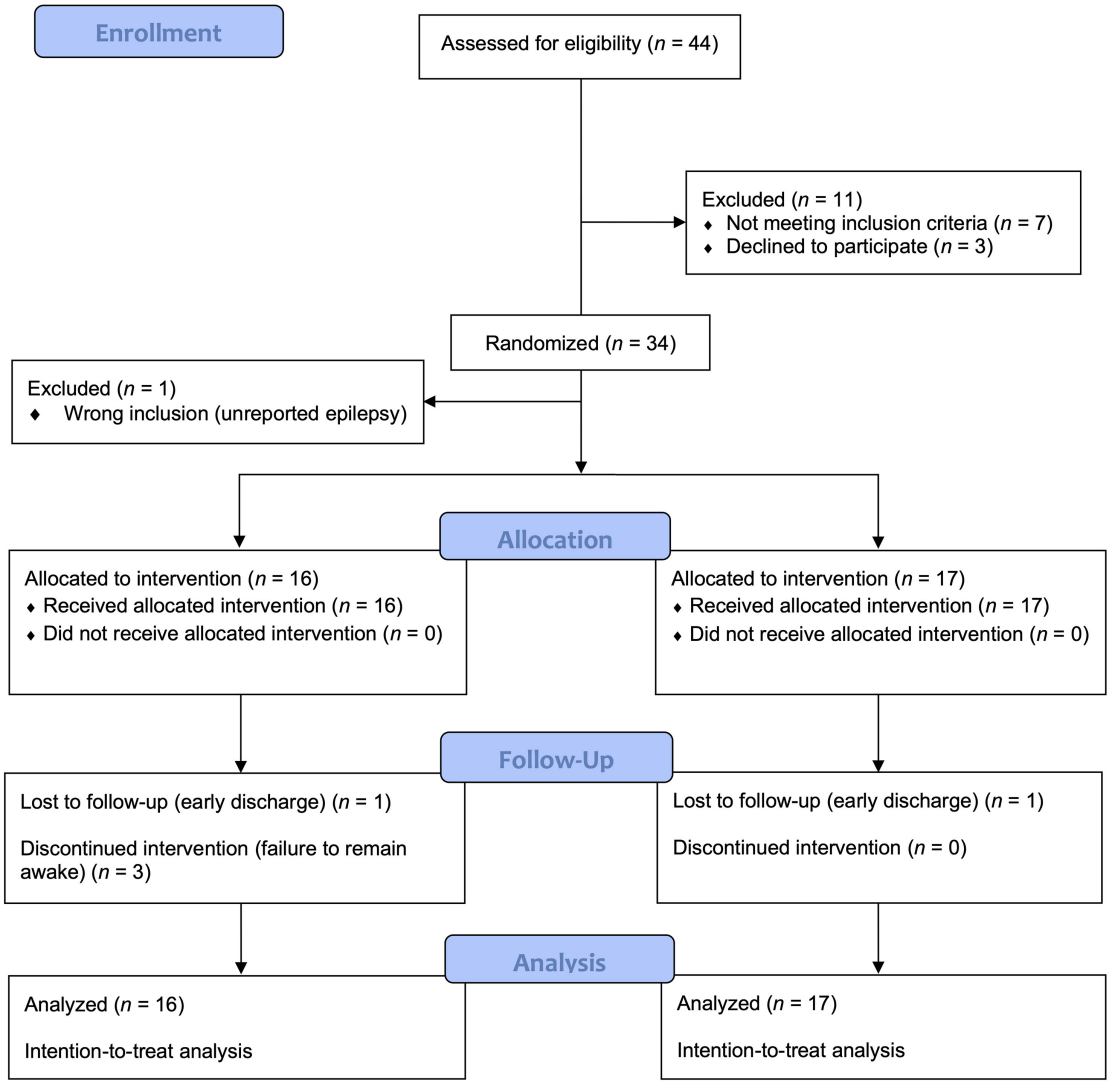
Patient recruitment and flow.

A total of 44 individuals were screened between August 2015 and June 2019, 33 of whom met the eligibility criteria and were randomized. The participants (26 women, 78.8%) had mean age (standard deviation, SD) 30.1 (10.9) years. Reasons for screening failure were uncertainty in diagnosis of depression (*n* = 4), ongoing withdrawal symptoms (*n* = 2), and psychotic symptoms at admission (*n* = 1). In addition, three patients declined participation during the screening process. One patient was excluded from the study after randomization due to an unreported diagnosis of epilepsy. Thus, sixteen patients in TSD/BLT group and seventeen in control group were included in the intention-to-treat analysis. In each group, one patient received the intervention but was lost to follow-up due to early discharge. Three participants in the TSD/BLT group discontinued the protocol due to failure to remain awake (Figure 1).

There were no statistically significant differences between the groups for the baseline demographic characteristics (Table 2). The mean (SD) MADRS-S total score at baseline was 36.8 (8.9), indicating high severity (36). Prior to psychiatric admission, 87.9% of the participants were clinician-evaluated at the emergency room with prominent suicidality defined as suicidal ideations, behavior, or attempt. Concurrent psychopharmacological treatment was similar in the two groups (Table 3).

**Table 2:** Baseline demographic and clinical characteristics (*N* = 33).

	**TSD + BLT group (*n* = 16)**	**Control group (*n* = 17)**	***p*** **-value**
**Sociodemographics**			
Age, years	31.3 (13.1) [18–54]	29.0 (8.6) [18–51]	0.561
Gender: female	12 (75.0)	14 (82.4)	
Married/live together	2 (12.5)	3 (17.6)	
**Suicidality at admission**			0.431
No suicidal ideations	1 (6.3)	3 (17.6)	
Suicidal ideations	4 (25.5)	7 (41.2)	
Suicidal ideations with specific plan/intention to act	6 (37.5)	4 (23.5)	
Suicidal attempt (actual, aborted, interrupted)/preparatory acts	5 (31.3)	3 (17.6)	
**Clinical measures at baseline**			
MADRS-S score	35.5 (9.8) [19–49]	38.0 (8.1) [24–54]	0.433
ISI score	16.7 (5.3) [8–27]	19.4 (6.2) [8–28]	0.185
CGI score	4.5 (0.7) [4–6]	4.2 (0.6) [4–6]	0.251
MEQ score	44.5 (10.7) [20–60]	42.4 (10.2) [27–60]	0.580
AUDIT score	6.4 (7.5) [0–22]	5.9 (4.5) [0–14]	0.821
DUDIT score	0.6 (1.4) [0–5]	1.6 (2.5) [0–7]	0.146
Major diagnosis			0.594
Major depressive disorder	10 (62.5)	13 (76.5)	
Mixed anxiety-depression disorder	4 (25.0)	2 (11.8)	
Bipolar disorder	2 (12.5)	2 (11.8)	
**Secondary diagnosis (at discharge)**			0.175
ADHD	3 (18.8)	0 (0)	
Anxiety disorder (including PTSD)	3 (18.8)	3 (17.6)	
Personality disorder	4 (25)	1 (5.9)	
**Somatic conditions**			
BMI	25.9 (5.4) [18–35]	25.1 (3.6) [19–31]	0.698
Diabetes	2 (12.5)	0 (0)	0.227
Hypertension or heart disease	4 (25)	1 (5.9)	0.175
Smoker	5 (31.3)	6 (35.3)	0.999
**Educational level**			0.934
Primary and lower secondary school	6 (37.5)	5 (31.3)	
Upper secondary school	5 (31.3)	5 (31.3)	
Technical college/short education	4 (25)	4 (25)	
Bachelor level or higher	1 (6.3)	2 (12.5)	
**Employment status**			0.34
Unemployed	0 (0)	6 (35.3)	
Student	4 (25)	1 (5.9)	
On sick leave	5 (31.3)	4 (23.5)	
Disabled (pension)	1 (6.3)	1 (5.9)	
Employed part- or full-time	6 (37.5)	5 (29.4)	

*Data are given as n (%) or mean (SD) [range]. ADHD, attention deficit hyperactive disorder; AUDIT, Alcohol Use Disorders Identification Test; BLT, bright light therapy; BMI, body mass index; CGI, Clinical Global Impression scale; DUDIT, Drug Use Disorders Identification Test; ISI, Insomnia Severity Index; MADRS-S, Montgomery-Åsberg Depression Rating Scale - Self Assessment; MEQ, Morningness-Eveningness Questionnaire; PTSD, post-traumatic stress disorder; SD, standard deviation; TSD, total sleep deprivation*.

**Table 3:** Mean daily dose of psychoactive medications by treatment group during study period. Combinations and changes in type or dose of agents.

**Drug/combinations and changes in type or dose of agent**	**TSD/BLT group (*n* = 16)**	**Control group (*n* = 17)**
**Antidepressants**		
Agomelatine, mg	50 (0) [1]	0 (0) [0]
Amitriptyline, mg	0 (0) [0]	87.5 (53.0) [2]
Bupropion, mg	200 (86.6) [3]	150 (0) [2]
Citalopram, mg	10 (0) [1]	20 (0) [1]
Clomipramine, mg	100 (0) [1]	0 (0) [0]
Duloxetine, mg	60 (0) [1]	0 (0) [0]
Escitalopram, mg	12.5 (6.5) [4]	12.5 (4.2) [6]
Fluoxetine, mg	30 (0) [1]	40 (20) [3]
Mirtazapine, mg	20 (8.7) [3]	30 (0) [4]
Paroxetine, mg	10 (0) [1]	0 (0) [0]
Sertraline, mg	143.8 (42.7) [4]	125 (28.9) [4]
Venlafaxine, mg	225 (0) [1]	150 (0) [2]
**In the study period treated with**		
No antidepressants	6.3 [1]	0 [0]
One type of antidepressant	56.3 [9]	64.7 [11]
Two types of antidepressant	37.5 [6]	29.4 [5]
Three types of antidepressant	0 [0]	5.9 [1]
**During the study period**		
Dose of antidepressants changed, % [*n*]	62.5 [10]	68.8 [11]
An antidepressant agent was added, % [*n*]	37.5 [6]	56.3 [9]
**Antipsychotics**		
Aripiprazole, mg	12.5 (3.5) [2]	0 (0) [0]
Olanzapine, mg	5 (0) [1]	2.5 (0) [1]
Quetiapine, mg	100 (0) [1]	75(0) [1]
Flupentixol, mg	1 (0) [1]	0 (0) [0]
Zuclopenthixol, mg	0 (0) [0]	25 (0) [1]
**Mood stabilizer**		
Lamictal, mg	212.5 (265.2) [2]	25 (0) [1]
Lithium, mmol	12 (0) [1]	18 (8.5) [2]
Topiramate, mg	100 (0) [1]	0 (0) [0]
Valproate, mg	0 (0) [0]	1,000 (0) [2]
**Sleeping agents**		
Zopiclone, mg	7.1 (1.0) [6]	8.8 (4.2) [8]
Zolpidem, mg	0 (0) [0]	10 (0) [1]
Melatonin, mg	2 (0) [3]	0 (0) [0]
Propiomazine, mg	37.5 (14.4) [4]	41.7 (13.0) [6]
**Others**		
Alimemazine, mg	40 (0) [1]	20 (0) [3]
Diazepam, mg	7.5 (3.5) [2]	10 (0) [1]
Hydroxyzine, mg	41.7 (28.9) [3]	33.3 (14.4) [3]
Oxazepam, mg	10 (0) [3]	13.8 (7.5) [4]
Promethazine, mg	75 (0) [1]	25 (0) [2]

*Data are given as mean (SD) [n] unless otherwise specified*.

### Primary Clinical Outcome

There was no numerical difference between groups with respect to clinical response (2 patients per group, *p* > 0.999) (Table 4).

**Table 4:** Major outcomes on intention-to-treat analysis.

	**Baseline (day 0)**		**Post-treatment (day 7)**		***p*** -value*
	**TSD/BLT group (*n* = 16)**	**Control group (*n* = 17)**	**TSD/BLT group (*n* = 16)**	**Control group (*n* = 17)**	
MADRS-S, mean (SD)	35.50 (9.79)	37.97 (8.05)	29.88 (10.52)	30.41 (10.39)	0.519
ISI, mean (SD)	16.69 (5.33)	19.41 (6.15)	12.38 (6.81)	15.0 (6.13)	0.959
CGI, mean (SD)	4.50 (0.73)	4.23 (0.56)	3.38 (0.96)	3.65 (0.61)	0.510
Response, *n* (%)	–	–	2 (12.5)	2 (11.7)	0.999
Remission, *n* (%)	–	–	1 (6.25)	0	0.485
ISI reduction ≥ 50%, *n* (%)	–	–	2 (12.5)	3 (17.64)	0.999
Length of stay, median (range)	–	–	16 (7-51)	22 (4-45)	0.820
Emergency room revisits, *n* (%)	–	–	2 (12.5)	5 (29.4)	0.245

* *For between group change vs. baseline. BLT, bright light therapy; CGI, Clinical Global Impression scale; ISI, Insomnia Severity Index; MADRS-S, Montgomery-Åsberg Depression Rating Scale - Self Assessment; SD, standard deviation; TSD, total sleep deprivation*.

### Secondary Outcomes

**Figure 2:**
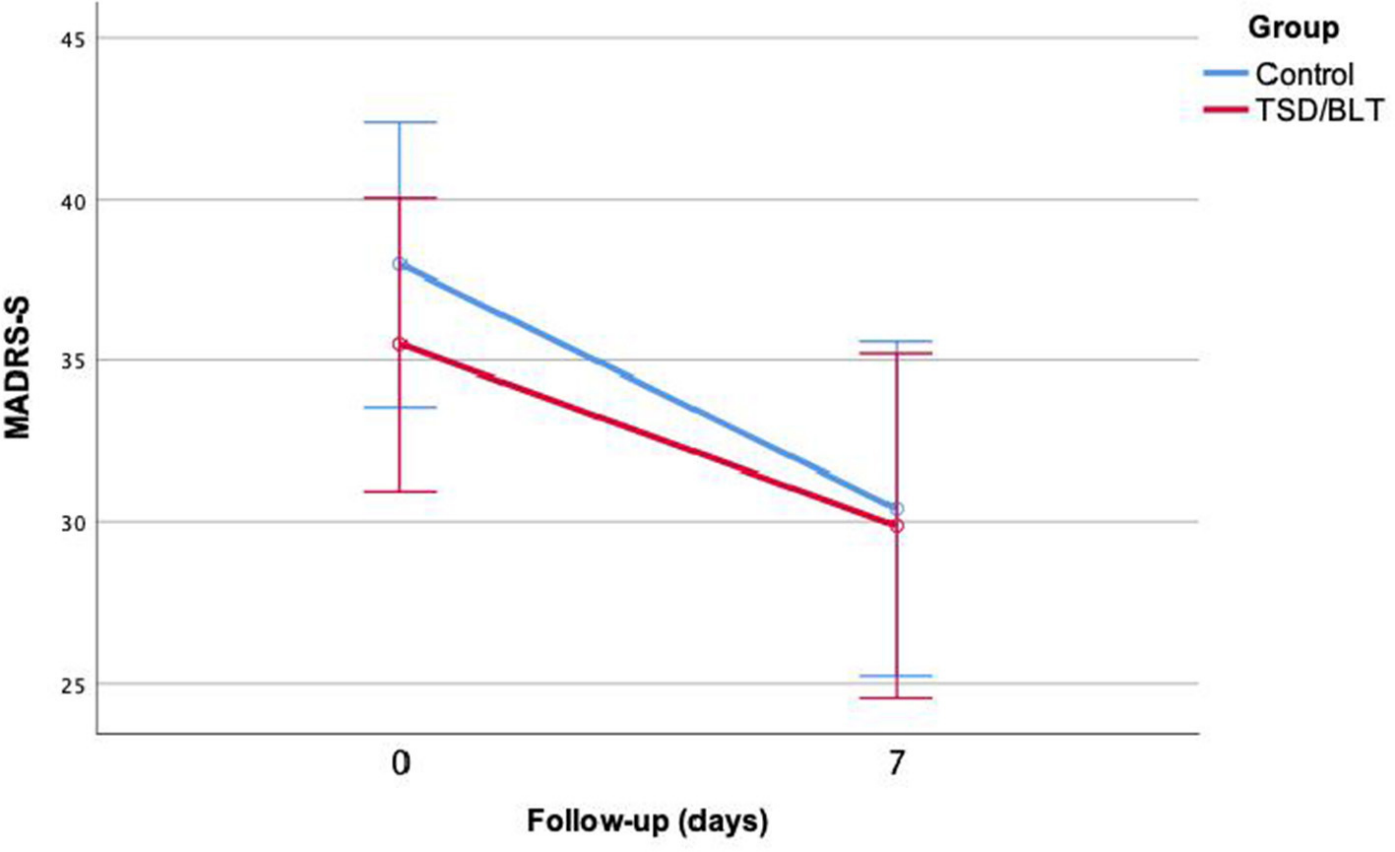
Estimated mean (95% confidence interval) MADRS-S score in the TSD/BLT and control groups before and after treatment.

One patient alone (in the TSD/BLT group) achieved remission. The TSD/BLT group did not show a significant reduction in the MADRS-S score after treatment [*t*(15) = 2.87, *p* = 0.12] in contrast to the control group which showed a significant reduction with a moderate effect size [*t*(16) = 3.43, *p* = 0.003, Cohen's *d* = 0.832]. There was no significant difference between the groups for MADRS-S score reduction [*t*(31) = −6.53, *p* = 0.519] (Figure 2).

Both groups showed a significant reduction in ISI score after treatment [*t*(15) = 3.616, *p* = 0.003 for TSD/BLT group and *t*(16) = 2.985, *p* = 0.009 for the control group]. However, there was no significant difference in the reduction of ISI score between the two groups [*t*(31) = −0.52, *p* = 0.959]. Five participants (2 in the TSD/BLT group and 3 in the control group) showed a ≥ 50% reduction in ISI score after treatment (*p* > 0.999).

Both the TSD/BLT [*t*(15) = 5.582, *p* < 0.001] and control [*t*(16) = 3.405, *p* = 0.004] groups showed significant reduction in CGI score after treatment, with no significant difference between groups [*t*(31) = 2.030, *p* = 0.51].

The mean duration from study enrollment until discharge was 18.3 days (median 16; range, 7–51) in the TSD/BLT group and 17.4 days (median 22; range, 4–45) in the control group (*p* = 0.820).

**Figure 3:**
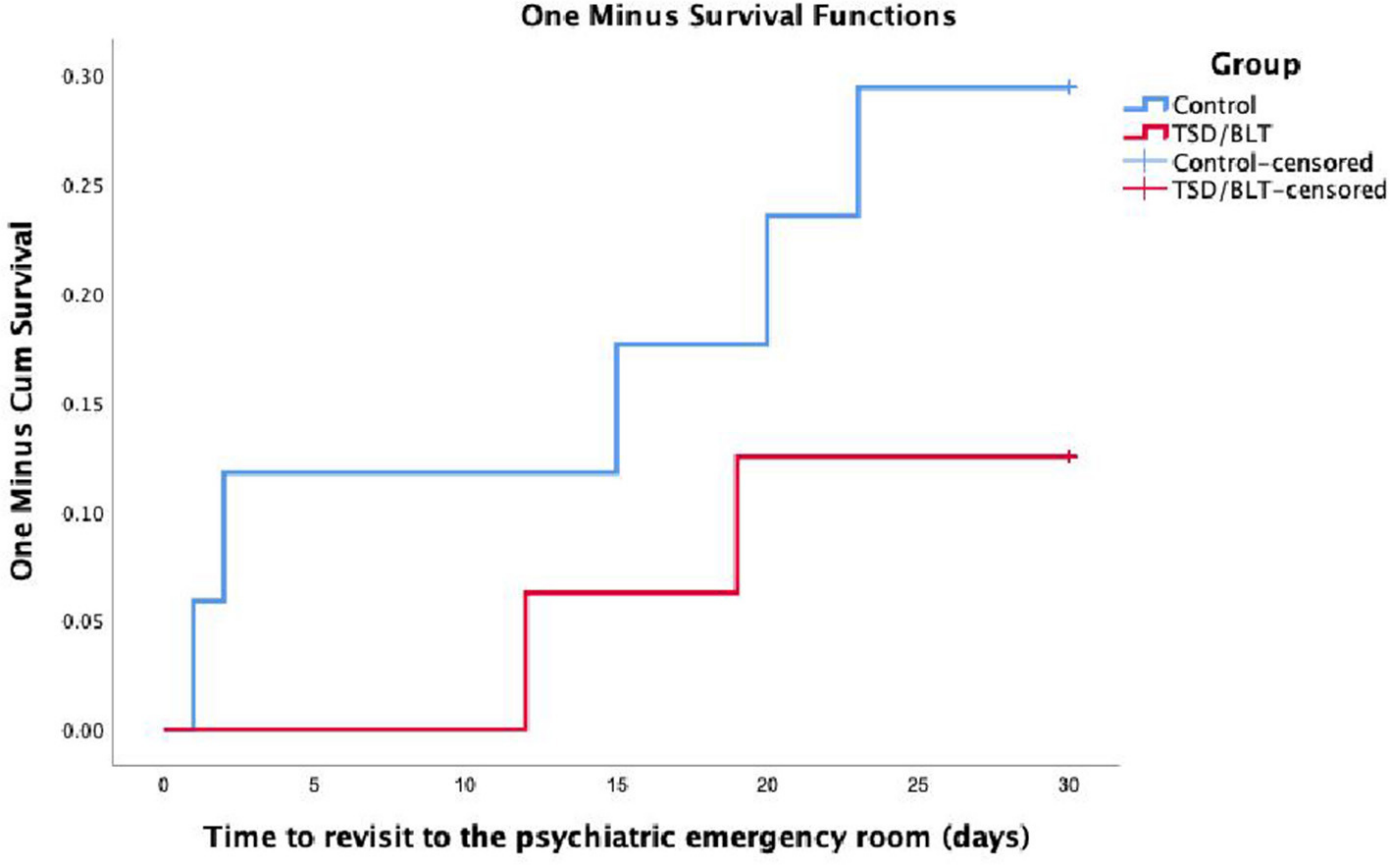
Kaplan-Meier survival curve for time to psychiatric emergency room revisit.

**Figure 4:**
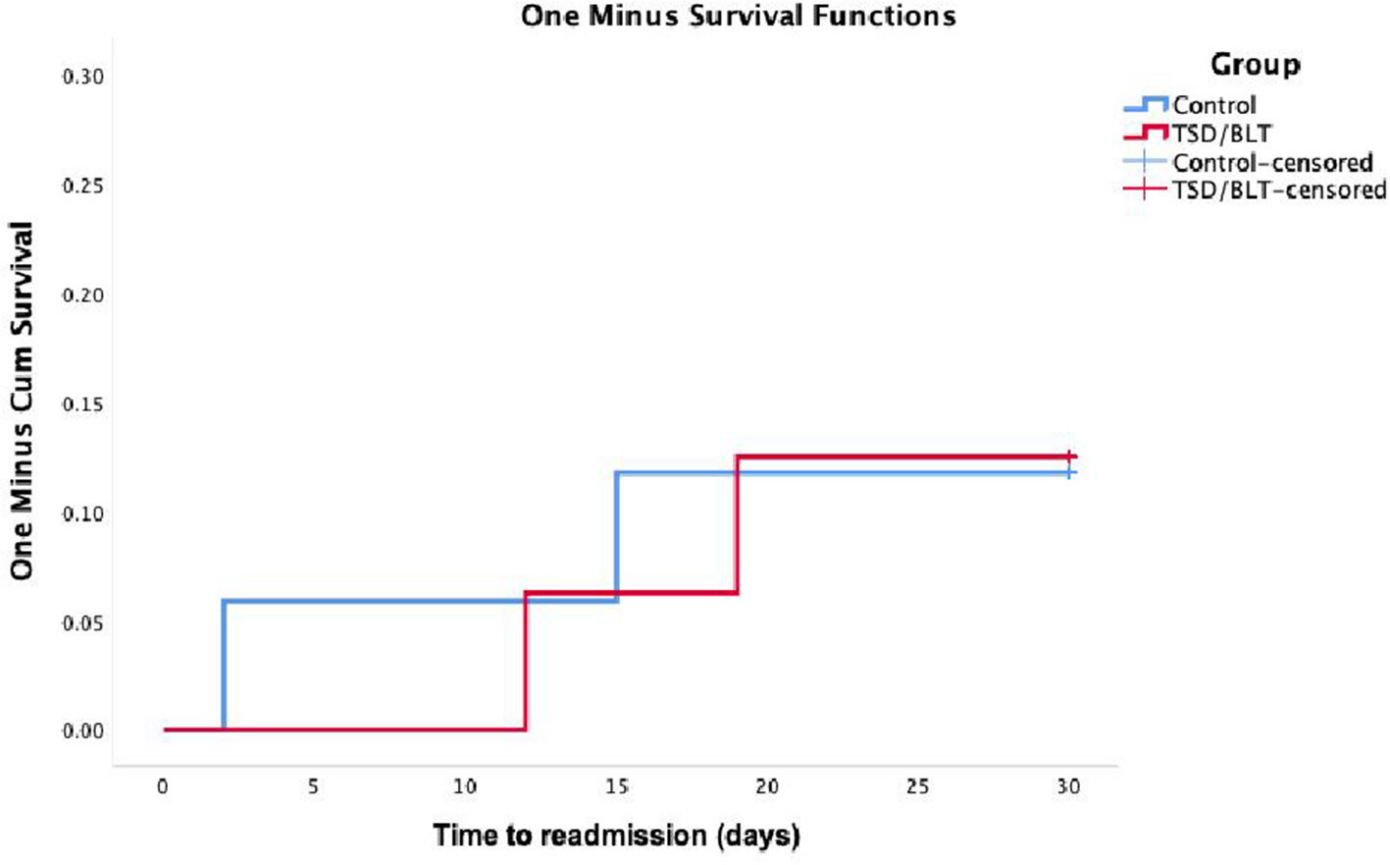
Kaplan-Meier survival curve for time to psychiatric ward readmission.

Thirty days after discharge, seven patients had revisited the emergency room due to mental distress/signs of relapse (12.5% of the TSD/BLT and 29.4% of the control group, Figure 3) and two patients from each group were readmitted to a psychiatric ward (Figure 4). In Kaplan-Meier analyses, log-rank test did not show significant differences in time to emergency room revisit (*p* = 0.245) or readmission to psychiatric ward (*p* = 0.975). There were no events in either group from day 30 to 90 after discharge.

### Self-Reported Patient Experiences

Thirteen participants from each group (81.3% in the TSD/BLT group and 76.5% in control group) filled in the questionnaire for evaluation of the add-on intervention received. The scale showed good internal consistency (Cronbach's alpha 0.745, mean inter-item correlation 0.337). Overall, both groups reported positive experiences of the treatment received without statistically significant differences between groups (Table 5).

**Table 5:** Self-report questionnaire for evaluation of acceptance of the received treatment.

Questionnaire items*	**TSD/BLT group (*n* = 13)**	**Control group (*n* = 13)**	***p*** **-value**
1. I was helped by the received intervention**	3.3 (1.3)	2.9 (1.3)	0.453
2. I experienced difficulties with the received intervention**	2.2 (1.4)	2.6 (1.6)	0.525
3. This feels like a modern way of treating mental disorders	4.4 (0.6)	3.4 (1.3)	0.22
4. I experienced this as coercive treatment/torture	1.2 (0.6)	1.2 (0.6)	0.999
5. If I could choose again, I would have consented to the treatment	4.2 (1.5)	4.1 (1.2)	0.999
6. I would recommend the treatment to a loved one with similar problems	4.2 (1.2)	3.3 (1.6)	0.113
7. This treatment should be available to patients who want it	4.8 (0.6)	4.7 (0.7)	0.558
Total score (item 2, 4 reversed)	29.5 (5.4)	29.6 (4.9)	0.172

*Data are given as mean (SD)*.

* *5-point symmetric agree-disagree scale (from 1 = “do not agree at all” to 5 = “fully agree”)*.

** *Either chronotherapeutic or psychoeducational intervention depending on the version of the questionnaire/group. BLT, bright light therapy; SD, standard deviation; TSD, total sleep deprivation*.

### Complications

Three patients (18.7%) discontinued sleep deprivation due to tiredness (*n* = 2), and increase in anxiety and self-harming thoughts during TSD (*n* = 1). There was no change in mean PANSS-5 score during TSD (0.19 ± 1.17, *p* = 0.53) or compared to the control group (*p* = 0.875). None of the patients showed signs of switch to mania or other serious adverse events.

In total, 50% of participants reported no adverse effects from TSD/BLT. The most frequently reported symptoms in the TSD/BLT group were tiredness (25%), worsening of insomnia (12%), eye strain (6%), restlessness (6%), and tonsillitis (6%).

### *Post* − *hoc* Analyses

Post-hoc analyses for the MADRS-S suicide item were conducted. Both the TSD/BLT [*t*(14) = 2.956, *p* = 0.01] and control [*t*(15) = 3.174, *p* = 0.006] groups showed statistically significant reduction in the MADRS-S suicide item. However, there was no significant differences between groups [*t*(29) = 0.010, *p* = 0.992].

## Discussion

The main objective of this pragmatic RCT was to investigate the effectiveness, acceptance, and feasibility of TSD combined with BLT as add-on to standard treatment for depression in a real-life clinical setting. Our study results did not disprove the null hypothesis as neither numerical nor statistical difference in the clinical response were found between the intervention and control groups. No statistically significant between-group differences were found regarding post-treatment depressive and insomnia symptoms, length of hospital stay, and readmission rate. The chronotherapeutic protocol was generally feasible without need of extra resources or costs, although recruitment difficulties occurred. One of the strengths of the current study was the assessment of patient experiences of chronotherapy, a previously understudied research area. Positive attitudes toward the chronotherapeutic intervention were reported, which was considered to be well-tolerated, although discontinuations occurred.

The studied treatment protocol was chosen based on previous promising results of the combination of TSD and BLT (25, 37, 38). It was further adjusted in consultation with the management and nursing staff of the psychiatric wards as part of the pragmatic design of our study. Certain treatment modalities may have played a role in the final outcome such as use of single instead of repeated TSD, initiation of BLT after recovery sleep instead of during TSD, and absence of standardized sleep management (e.g., SPA, STS). Direct comparison of these modalities in a clinical trial has not been performed to our knowledge, although repeated TSD, initiation of BLT during TSD, and SPA may be more favorable (14).

Despite differences in treatment protocols, our findings are in line with a RCT among inpatients with moderate to severe depression (39). The study by Kragh et al. (39) evaluated an add-on chronotherapeutic protocol to standard treatment (3 nights TSD within 1 week, BLT, and STS) which was previously found to be effective for both short- and long-term outcomes in an outpatient population (35, 40, 41). When applying the chronotherapeutic protocol to inpatients, no statistically significant differences were found between intervention and control groups in response and remission rates. A transient effect of chronotherapy on depressive symptoms was noticed 1 week after treatment (and in temporal proximity with the third TSD), which was mainly explained by the sleep items of the Hamilton Depression Rating Scale (HDRS). The HDRS contains more sleep and psychomotor items than MADRS-S and its validity has been extensively criticized (42, 43). Moreover, another RCT evaluated 1-night TSD as add-on to BLT in juvenile depressed inpatients (44). No additive effect of TSD on sleep quality or on depressive symptoms was found. Meta-analyses on TSD have shown divergent conclusions on its efficacy, primarily explained by differences in inclusion criteria (10–12, 45–47). To our knowledge, only one study has previously investigated patient experiences of chronotherapy in patients with depression, reporting positive attitudes in line with our results (48). Overall, our study results support the thesis that TSD followed by BLT is a safe, inexpensive, and well-tolerated treatment option for depressed inpatients. Even if TSD is to be found to only have a rapid but transient effect, it may still have a place in the therapeutic arsenal against acute depressive states. For instance, TSD could show patients the malleability of depressive symptoms, thereby, improving self-efficacy as Kragh et al. have suggested (39).

Controlled trials on TSD are generally challenging due to the lack of blinding and obvious control condition. Namely, there is no appropriate sham alternative which can be used in the control group. Most of the RCTs have used an add-on design (i.e. only standard treatment in the control group) which is, however, more vulnerable for bias. To address this matter, other control conditions such as low-intensity exercise and mild sleep/light management has been previously used (39, 40, 49). A short sleep hygiene consultation was chosen as control condition as it was in line with the pragmatic design of the study. Namely, focus on sleep hygiene is common at a self-care and primary care level in Sweden. Moreover, the consultation took place in one short session and emulated regular clinical advice. Thus, it didn't qualify as a sleep hygiene education program although their context, length, administration method may vary (50). A strength in using such methods as comparators is the low efficacy compared to other interventions (50, 51).

In depression research, high risk of suicide, psychotic symptoms, or treatment-resistant depression are factors that have traditionally been used to exclude patients from clinical trials, leaving a gap in the knowledge of acute management in clinical settings (52, 53). With our pragmatic design, we aimed to evaluate TSD in a representative sample of the actual real-world inpatient population. Consequently, the underlying diagnoses varied in the sample despite the high depression severity. In contrast to previous RCTs on TSD, suicidal patients were included and were highly represented (87.9 %), thus increasing the generalizability of the findings. Namely, that the acute management of suicidality during hospitalization is paramount, especially in depressed patients (54).

Conducting clinical trials in real-world health system practice can be challenging, especially in psychiatry (18). Despite the high relevance of the research question and the simplicity of the study protocol, the anticipated recruitment target was not reached. The close-down of two of four psychiatric wards as well as the COVID-19 pandemic extensively impacted the ability to recruit patients. Lack of time and resources devoted to research are common barriers to patient recruitment into RCTs and our study was not an exception (55). Moreover, intellectual and emotional challenges arise when combining research with clinical roles, subsequently affecting recruitment, as has also been previously reported (56).

Beyond the pragmatic setting, special focus was given to the examination of the rapid effect of TSD/BLT when designing the study. All major outcomes were measured for a 1-week period and in temporal proximity to hospital admission. Nonetheless, the definition as well the optimal method and time-frame to evaluate rapid antidepressant actions have varied across the literature, without clear consensus (1). Characteristically, the definition of rapid onset of antidepressant effects ranges from significant response rates within a few hours to 20% symptom reduction up to 2 weeks (57).

Future studies on TSD need to identify feasible and effective chronotherapeutic protocols in depression in real-world health system practice. Effectiveness-implementation hybrid designs may be a valuable approach considering the lack of pragmatic trials and implementation of chronotherapy in daily clinical practice (58). Repeated TSD and/or standardized sleep management (e.g., SPA, STS) may be important modalities and head-to-head comparisons are warranted. Also, other treatment modalities such as light spectrum/intensity and exposure time may play a role in the efficacy of combined chronotherapy (59, 60). Eligible subgroups of responders as well the relevance of individual adaptation of treatment modalities needs to be further examined. Bipolar disorder, low levels of concurrent anxiety, previous response to antidepressants, positive diurnal variation (mood best in the evening), and evening chronotypes may predict better response to chronotherapeutic interventions (13, 14, 41, 61). However, their predictive relevance needs to be investigated in larger samples.

## Limitations

Despite our efforts we did not meet our anticipated recruitment target. Thus, a major limitation of this study is the relatively small sample size in relation to our prior power analysis. However, the probability of type II error should be considered very low with regard to the observed numerical difference (i.e., the control group showed statistically significant and higher symptom reduction than the TSD/BLT group). Moreover, recent studies on combined chronotherapy have reported lower response rates (20, 39, 44, 58) than the presumed average of 50% (45) with the exception of two cohort studies (22, 62). Both groups showed improvement at week 1. Another control group without any intervention could have been informative on the additive effect of the sleep hygiene consultation, if any, since this was a minimal intervention emulating common clinical advise.

No data on the eligibility assessment were available as pre-screening was conducted by the physicians in charge. It is therefore possible that potentially eligible patients were not asked to participate in the study. This might present a selection bias of patients deemed to have better compliance to the study protocol, limiting the generalizability of the drop-out rate results. However, this is unlikely to affect the results of effectiveness, since, in a clinical setting, patients are generally offered feasible treatment options. The follow-up period was short (1 week) but relevant to the aims of the study and the average length of hospital stay for depression in Sweden (63). Besides, imminent measurement errors (e.g., regression to mean) and other confounders should be evenly distributed between the groups through randomization. However, the sample was less than the general rule of 50 participants (64).

The measures of depressive and insomnia symptoms were self-reported. Although the choice was consistent with the pragmatic design, the question of data validity is raised. Nonetheless, the self-rated version of MADRS has previous shown a high correlation with the clinician-rated scale (65, 66). Moreover, ISI has been shown to be a valid and sensitive tool to detect changes in perceived sleep difficulties with treatment (30) and eligible for use in patients with affective disorders (67, 68). Nevertheless, additional clinician-rated measures would have been interesting as discrepancy between subjective and objective severity of the depressive symptoms may have a predictive value in chronotherapeutic interventions (69).

In general, unintentional microsleep episodes (i.e., short phases of sleepiness) may occur during sleep deprivation. The risk of microsleeps was not fully monitored in our study. However, self-reporting and nurse-monitoring may not be adequate detection methods for microsleeps (70). Additionally, more stringent adherence and monitoring strategies (such as electroencephalography) would not be compatible with the pragmatic design of the study.

## Conclusion

In conclusion, a single night of total sleep deprivation followed by 6 days bright light therapy was not effective as a rapid relief for depression at 1-week follow-up. Inpatients with severe suicidal ideation may not be benefit from add-on combined chronotherapy despite the treatment's positive acceptability. Future studies need to identify feasible and effective chronotherapeutic protocols in in a real-life clinical setting as well as potential subgroups of responders.

## Data Availability Statement

The raw data supporting the conclusions of this article will be made available by the authors, without undue reservation.

## Ethics Statement

The studies involving human participants were reviewed and approved by University of Gothenburg Ethics Committee (No. Ö11-2014). The patients/participants provided their written informed consent to participate in this study.

## Author Contributions

ZS and SS were involved in the conceptualization and supervision of the study. ZS and MW-J developed the methodology. MI, ZS, MW-J, GV, and CK were involved in the conduct of the study. MI, GV, and CK curated the study data. MI and SS conducted the formal analysis. MI undertook administration of the project and wrote the first draft of the article. All authors were involved in review, editing of the article, and approved the submitted version.

## Conflict of Interest

SS's research time was financed by grants from the Swedish state under the agreement between the Swedish government and the county councils, the ALF agreement (ALFGBG-786541). The funder had no influence over the conduct or reporting of the study. The remaining authors declare that the research was conducted in the absence of any commercial or financial relationships that could be construed as a potential conflict of interest.

## Publisher's Note

All claims expressed in this article are solely those of the authors and do not necessarily represent those of their affiliated organizations, or those of the publisher, the editors and the reviewers. Any product that may be evaluated in this article, or claim that may be made by its manufacturer, is not guaranteed or endorsed by the publisher.
